# Analysis of the diagnostic value of CD138 for chronic endometritis, the risk factors for the pathogenesis of chronic endometritis and the effect of chronic endometritis on pregnancy: a cohort study

**DOI:** 10.1186/s12905-016-0341-3

**Published:** 2016-09-05

**Authors:** Yu-qing Chen, Rui-li Fang, Yuan-na Luo, Can-qiao Luo

**Affiliations:** 1 Department of Gynecology and Obstetrics, First Affiliated Hospital, Sun Yat-sen University, Guangzhou, Guangdong Province People’s Republic of China; 2 Department of Gynecology and Obstetrics, Shenzhen Luohu People’s Hospital, Shenzhen, Guangdong Province People’s Republic of China; 3 Medical Records and Statistics Room, First Affiliated Hospital, Sun Yat-sen University, Guangzhou, Guangdong Province People’s Republic of China; 4 Department of Pathology, First Affiliated Hospital, Sun Yat-sen University, Guangzhou, Guangdong Province People’s Republic of China

**Keywords:** Chronic endometritis, CD138, Infertility

## Abstract

**Background:**

To investigate the role of CD138 immunohistochemistry in the diagnosis of chronic endometritis (CE) and the risk factors for assisted conception patients having CE complications.

**Methods:**

Ninety-three patients, with normal uterine shape confirmed by examination and who were planning to undergo assisted conception treatments, were selected as research subjects. Endometrial tissue was isolated for routine hematoxylin and eosin (HE) and CD138 immunohistochemical staining. Additionally, the disease histories of patients were collected, and the reproductive prognosis was followed up.

**Results:**

① CE detection rate: The rate of CD138 immunohistochemical staining was greater than that of HE staining (27.96 % vs. 26.89 %, *P* <0.05); ② Pregnancy rate: the pregnancy rate of CD138-positive patients (7.7 %) was lower than the pregnancy rate of CD138-negative patients (31.3 %) (*p* = 0.017 < 0.05); ③ The results from univariate analysis showed that a previous history of prolonged menstrual bleeding episodes, an abortion history, and complications of fallopian tube obstruction were associated with CE (*P* <0.05). The results of logistic regression analysis confirmed that prolonged menstrual bleeding episodes (*P* = 0.014, OR = 5.394, 95 % CI 1.405-20.699), a previous abortion history (*P* = 0.029, OR = 3.194, 95 % CI 1.125-9.073), and fallopian tube obstruction (*P* = 0.028, OR = 3.274, 95 % CI 1.139-9.415) were independent risk factors for positive CD138 results.

**Conclusions:**

CD138 immunohistochemistry can improve the CE diagnosis rate. A previous history of prolonged menstrual bleeding episodes, an abortion history, and a history of fallopian tube obstruction are risk factors for chronic endometritis, and a CD138 immunohistochemical examination should be advised among them.

## Background

Infertility is a relatively common gynecological disease whose incidence has increased in recent years. Relevant studies have shown that the incidence of chronic endometritis (CE) in infertile patients ranges from 0.2–46 % [1, 2]. Infertility and natural abortion may be associated with and CE [1, 3, 4]. Recent studies pointed out that the prevalence of CE was 14 % in patients with recurrent implantation failure (RIF) after IVF [5] and women with CE had lower implantation rates (11.5 %) in the IVF cycle following treatment than did those with CE absent (32.7 %) [6]. Furthermore, *Cicinelli et al.* demonstrated that CE is a common finding in women complaining of repeated implantation failure and appropriate antibiotic treatment had improved significantly the rate of successful pregnancies in women without signs of CE compared with women who were with persistent CE [7]. The reports suggest the presence of CE may alter endometrial receptivity and negatively affect fertility outcome.

However, CE normally does not show symptoms and is easily overlooked in clinical practice [2, 4, 8]. The diagnosis of CE is based on the detection of abnormal plasma cell infiltration in the endometrial stroma [9] (Kasius et al., 2011). Using conventional hematoxylin and eosin (HE) staining, it is sometimes difficult to distinguish the plasma cells from the fibroblasts and monocytes of the endometrial stroma; thus, the success rate of accurate diagnosis and treatment of CE is not high [9, 10]. The transmembrane heparin sulfate proteoglycan syndecan-1 (CD138) is a syndecan, which is a specific marker of plasma cells. Therefore, in current clinical practice, CD138 immunohistochemistry is used for the detection of chronic endometritis to improve its diagnosis rate [11]. However, because CE tends to be overlooked in clinical practice, and clinicians have a limited understanding of the association between CE and infertility, this method is rarely used for further examination. The present study has compared the detection rate of chronic endometritis by HE and CD138 immunohistochemical staining and investigated the difference in sensitivity between CD138 immunohistochemical staining and conventional HE staining in the diagnosis of chronic endometritis, while further analyzing the relevant influential factors for the pathogenesis of chronic endometritis to provide a reference for clinical treatment.

## Methods

### General information

We prospectively collected information from 93 patients who recently had a laparoscopy and hysteroscopy examination and planned to undergo assisted conception treatment at the First Affiliated Hospital of Sun Yat-sen University between April 2013 and December 2013 due to infertility. The ages of the patients were between 23 and 43 years, with an average age of 34.36 ± 4.63 years. The average time of pregnancy was 1.3 ± 1.3, and the average time of giving birth was 0.50 ± 0.56. None of the 93 assisted conception patients had chronic abdominal pain or lumbosacral pain. Laparoscopy examinations confirmed that 43 patients had unilateral fallopian tube obstruction, and one patient had bilateral fallopian tube obstruction. Of these patients, 12 patients had prolonged menstrual bleeding episodes, 32 patients had undergone abortion surgery, 12 patients had a pelvic inflammatory disease history, 6 patients had history of cervical mycoplasma infection, 6 patients had history of cervical chlamydial infection and 12 patients had complications related to low sperm counts in male partners. A prolonged menstrual bleeding episode refers to a prolonged menstrual period or bleeding between menstrual cycles.

The case inclusion criteria consisted or patients who ① were married, ② had a regular sexual life for longer than one year without contraception that did not result in pregnancy, ③ were clinically diagnosed with fallopian tube-factor infertility, male factor infertility, or infertility for an unknown reason.

Case exclusion criteria included patients who had ① complications with other intrauterine lesions, such as intrauterine adhesions, submucosal uterine fibroids, uterine septum, and other uterine malformations; ② complications of uterine fibroids, endometriosis, ovarian tumor, or hydrosalpinx; ③ female hormone abnormalities; ④ vaginitis found by vaginal leucorrhea examination (including bacterial vaginosis or vulvovaginal yeast vaginosis or trichomonas vaginitis), pelvic tenderness during gynecological examination, or suspected vaginitis or pelvic inflammatory disease or acute infection period.

The present study has been reviewed and approved by the Ethics Committee ([2014] 64) of the First Affiliated Hospital of Sun Yat-sen University, and an informed consent was signed on by the patients involved.

### Specimen collection

All patients underwent laparoscopic surgery during the early follicular phase. After anesthesia, an endometrium biopsy was collected before the hysteroscopic and laparoscopic surgery was commenced.

### Processing of the pathological specimens of the endometrium

After the tissue was collected, it was immediately immersed in 10 % neutral formaldehyde for fixation. After ethanol gradient dehydration, the tissue was cleared with xylene. The tissue was immersed in paraffin for embedding, and 4 μm serial sections were cut. After HE staining, immunohistochemical staining was performed. Immunohistochemistry was performed by following the detailed procedures of the kit (DAKO, Carpinteria, CA, USA).

The pathological results were independently analyzed by two experienced pathologists to detect plasma cells for diagnosis. In the case of a disagreement, the obtained results were discussed by the two pathologists. If necessary, a third pathologist was consulted to resolve a dispute.

**Fig. 1:**
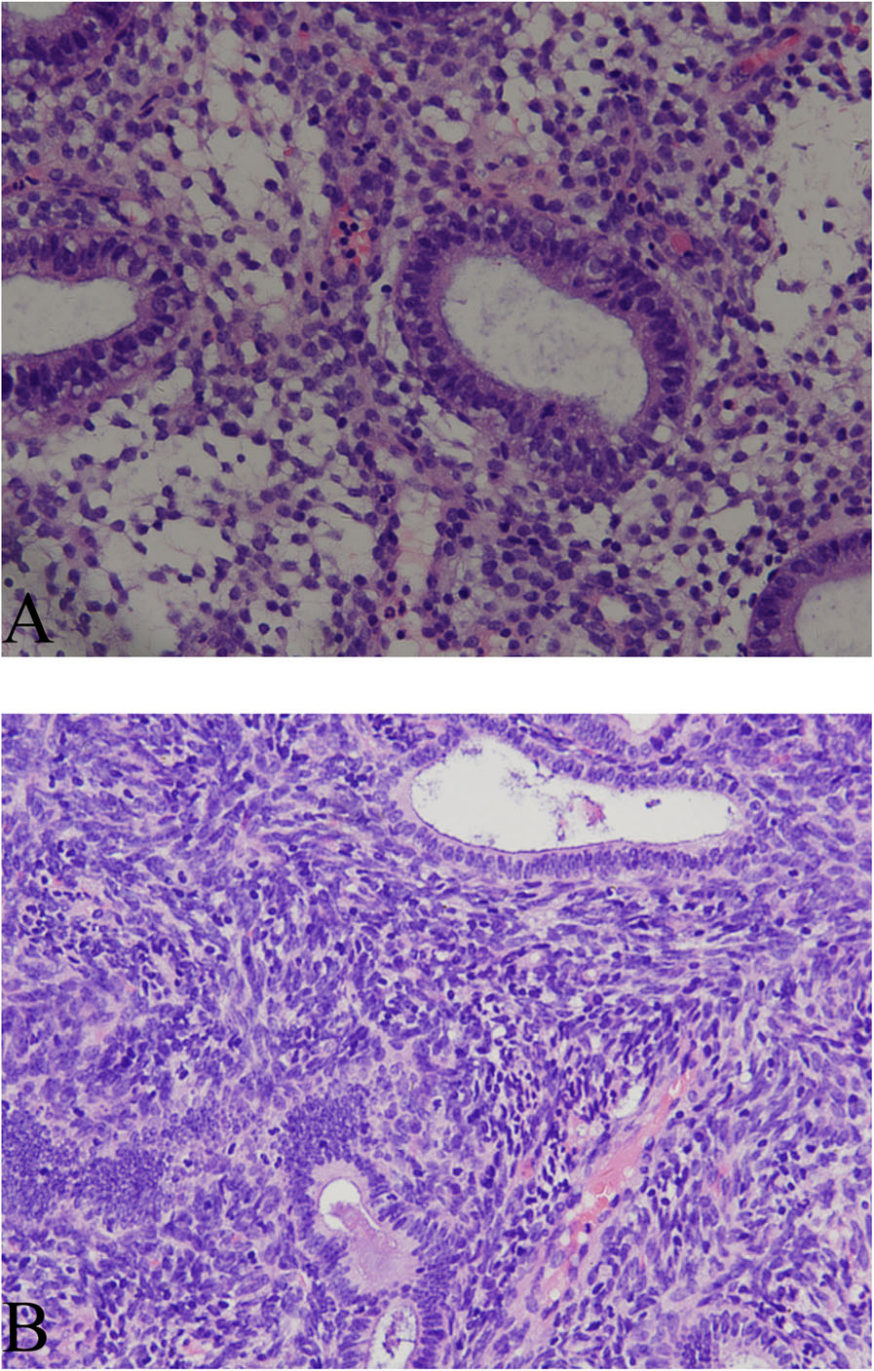
HE staining performance of suspected chronic endometritis (**a**) and non-chronic endometritis (**b**) (HE, ×200)

Pathological diagnostic criteria for chronic endometritis [9, 12]: For HE-stained specimens, the criteria consisted of the following: ① For the diagnosed chronic endometritis group, typical plasma cells were visible in the endometrial stroma. ② For the suspected chronic endometritis group, no plasma cells were detected in the HE-stained endometrium, and endometrial stromal cells showed spindle-like shapes similar to fibrosis and lymphocytes that were clustered focally in endometrial stromal cells (Fig. 1a). ③ For the non-chronic endometritis group, neither of the first two conditions were met in HE-stained specimens (Fig. 1b).

**Fig. 2:**
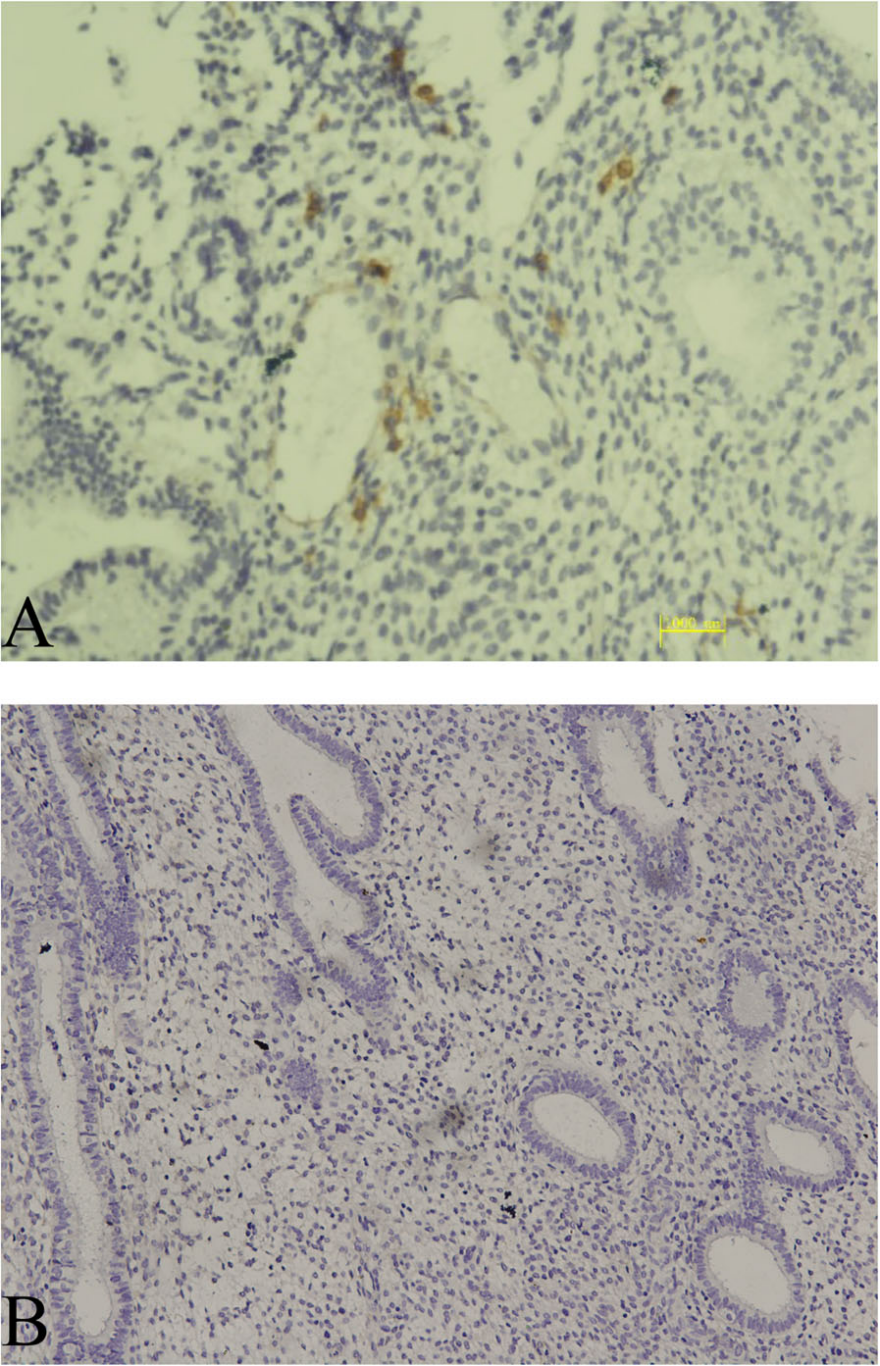
Positive staining of CD138 in chronic endometritis (**a**) and negative staining of CD138 in non-chronic endometritis (**b**)

The criteria for CD138 immunohistochemically stained specimens [9, 12]: After CD138 immunohistochemistry staining, the plasma cell membrane showed strong positive staining, while the cytoplasm showed weak positive staining. Under microscopic observation, the nucleus was rounded but located on one side of the cell, and the thick chromatin was radially arranged along the nuclear membrane to form a wheel shape (Fig. 2a). The cytoplasm was basophilic. ① For specimens diagnosed as chronic endometritis, in each 400 x magnification field, five or more typical plasma cells were observed in the endometrial stroma. ② For specimens not diagnosed as chronic endometritis, no plasma cells were observed in CD138 immunohistochemically stained specimens, or less than five plasma cells were observed in each 400x magnification field (Fig. 2b).

### Follow-up examinations

All patients underwent at least 12 months of follow-up after the operation; the interval of follow-up evaluations was three months. The information needed to calculate the cumulative pregnancy rate of all patients was collected through phone calls and outpatient visits.

### Statistical analysis

All data were analyzed with the SPSS 13.0 statistical software package. Measured data are represented by means ± standard deviations (*x* ± *s*). If the data of two samples were normally distributed and the two population variances were equal at the significant level, two independent samples *t* test analysis was used. For data that did not have a normal distribution or were not equal at the significant level, a Wilcoxon rank-sum test was used. A Chi-square test was used for the cell count data. For data that did not meet the requirement for a Chi-square test, Fisher’s exact test was used. Pearson X2 independence test was used for the association analysis of categorical variables. All variables with a value of *p* < 0.05 in the univariate analysis were included in the multivariate unconditional logistic regression model by the stepwise forward method. A value of *P* < 0.05 indicated statistical significance.

## Results

### The difference in the positive staining rate between the HE group and the CD138 immunohistochemistry staining group

A Chi-square test was used to compare the positive rate of the two groups (Table 1). The positive rate of the HE group was 0, and the suspected positive rate was 26.89 % (25/93). Both rates were lower than the positive rate of the CD138 immunohistochemistry group (27.96 %, 26 / 93), and the difference was statistically significant (*P* = 0.041). According to CD138 immunohistochemical results, the 93 patients were divided into two groups, the chronic endometritis group (CE, *n* = 26) and the non-chronic endometritis group (NCE, *n* = 67). The clinical data of the two groups are shown in Table 2. Except for a significant difference in oviduct blockage between the two groups (*P* = 0.030), the differences of all other variables were not statistically significant (*P* > 0.05).

**Table 1:** The comparison of diagnosis rate for CE between HE and immunohistochemical staining of CD138

		CD138			
		Positive	Negative	Totality	
HE	Positive	0	0	0	
	Probable positive	3	22	25	*P* = 0.041
	Negative	23	45	68	
	totality	26	67	93

**Table 2:** The comparison of the general conditions

	CE (*n* = 26)	NCE (*n* = 67)	*t/X* ^*2*^ */Z*	*P*
Age (y)^1)^	35.50 ± 3.89	33.92 ± 4.84	−1.515	0.130
Body mass index (kg/m^2^)^1)^	21.50 ± 1.54	21.31 ± 3.42	−0.767	0.443
Menstrual Cycle (day)^1)^	28.36 ± 2.24	29.40 ± 3.93	−0.666	0.505
menstrual period (day)^1)^	5.57 ± 1.28	5.22 ± 1.70	−1.007	0.314
Gravidity (n)^1)^	1.29 ± 1.18	1.26 ± 1.36	−0.276	0.782
Parity (n)^1)^	0.38 ± 0.48	0.54 ± 0.58	−1.104	0.270
infertility duration (y)^1)^	3.42 ± 1.47	3.33 ± 1.31	−0.630	0.529
Primary infertility (n)^2)^	9 (34.6)	28 (41.8)	0.403	0.526
male asthenospermia and oligospermia (n)^2)^	6 (23.1)	6 (9.0)	3.324	0.068
Tubal blockage (n)^2)^	17 (65.4)	27 (40.3)	4.729	0.030*

1. CE: Patients with chronic endometritis; NCE: patients without chronic endometritis

2.^1)^Mann–Whitney u Test ^2)^Chi-square test

3.*Significant difference at *p* < 0.05

### The effect of negative and positive CD138 results on the implantation of fertilized eggs in infertile patients

All 93 patients underwent assisted pregnancy treatment at an opportune time after surgery (IVF-ET and five cases of artificial insemination). And the five patients were all from the non-chronic endometritis group. Twenty-three patients successfully became pregnant after the operation, 20 patients underwent IVF-ET assisted procedures, and three patients received artificial insemination. The pregnancy rate of CD138 negative patients (31.3 %, 21/67) was higher than that of CD138 positive patients (7.7 %, 2/26), and the difference was statistically significant (*P* = 0.017). The results are shown in Table 3. In addition, we independently evaluated the pregnancy rate of IVF cycles and the results were significantly different (*P* = 0.048).

**Table 3:** The related risk factors for chronic endometritis

factors	Totality n	CE (*n* = 26)	NCE (*n* = 67)	*X2*	*P*	*r*
prolonged menstrual bleeding						
Yes	12	8	4	10.25	0.003*	0.315
No	81	18	63			
delivery history						
Yes	42	9	33	1.621	0.203	0.131
No	51	17	34			
previous abortion history						
Yes	32	14	18	6.042	0.014*	0.247
No	61	12	49			
history of spontaneous abortion						
Yes	14	6	8	1.817	0.178	0.138
No	79	20	59			
history of vaginitis						
Yes	51	15	36	0.119	0.73	0.036
No	42	11	31			
history of pelvic inflammation						
Yes	12	6	6	3.324	0.068	0.186
No	81	20	61			
History of cervical mycoplasma infection						
Yes	6	3	3	1.547	0.214	0.128
No	87	23	64			
History of cervical chlamydial infection						
Yes	6	3	3	1.547	0.214	0.128
No	87	23	64			
Tubal blockage						
Yes	44	17	27	4.729	0.030*	0.22
No	49	9	40			
pregnancy outcome						
pregnancy	23	2	21	5.629	0.017*	−0.246
unpregnancy	70	24	46			

*Significant difference at *p* < 0.05

### Analysis of CD138 positive risk factors

The clinical data of CD138 positive patients were retrospectively analyzed. Pearson’s *X* ^*2*^ independence test was applied to each possible factor of CD138 positive patients to perform an association analysis of categorical variables (Table 3). The results showed that prolonged menstrual bleeding episodes (*χ* ^*2*^ = 10.250, *P* <0.05, *r* = 0.315), a previous abortion history (*χ* ^*2*^ = 6.042, *P* <0.05, *r* = 0.247), fallopian tube obstruction (*χ* ^*2*^ = 4.729, *P* <0.05, *r* = 0.220) were associated with CE. Moreover, these factors were used as independent variables, and CD138 was used as the dependent variable in multivariate logistic regression analysis. The results confirmed that prolonged menstrual bleeding episodes (*P* = 0.014, *OR* = 5.394, 95 % *CI* 1.405-20.699), a previous abortion history (*P* = 0.029, *OR* = 3.194, 95 % *CI* 1.125-9.073), and fallopian tube obstruction (*P* = 0.028, *OR* = 3.274, 95 % *CI* 1.139-9.415) are independent risk factors of positive CD138 results (Table 4).

**Table 4:** Logistic regression analysis for chronic endometritis

Variable	*B* value	*P* value	Odds ratio	95 % *CI*
prolonged menstrual bleeding	1.685	0.014*	5.394	1.405–20.699
previous abortion history	1.161	0.029*	3.194	1.125–9.073
Tubal blockage	1.186	0.028*	3.274	1.139–9.415

*Significant difference at *p* < 0.05

## Discussion

Infertility is a common gynecological disease. The endometrium is the location for fertilized egg implantation, and chronic endometritis may cause damage to endometrial receptivity, resulting in infertility or miscarriage. Relevant studies [4] have shown that the implantation rate of embryo transfer in infertile patients complicated with chronic endometritis is significantly lower than that of infertile patients without endometritis. Erika B et al. [5] also found a higher failure rate of implantation into the endometrium in patients with chronic endometritis. The preventive use of antibiotics in patients with repeated failure of implantation and recurrent miscarriages can effectively improve their reproduction prognosis [4, 5]. Similarly, Cicinelli E et al. further confirmed the results in the recent study [7]. If the chronic endometritis is not treated, the success rate of natural conception and artificial insemination will be reduced, and these processes may even result in adverse obstetric complications [13]. Kotaro Kitaya et al. [4] analyzed and labeled syndecan-1 via immunohistochemical methods to identify stromal plasma cells. These researchers determined that among patients with habitual abortion, approximately 10 % of the patients suffered from chronic endometritis, while in habitual abortion patients without a known cause, the prevalence of endometritis was approximately 12–13 %. All patients with a chronic endometritis disease history had a miscarriage history during the first three months of pregnancy. This finding indicates that chronic endometritis is a factor for habitual abortion that should not be ignored. The results from the present study have shown that among infertile patients, the detection rate of chronic endometritis is 27.96 %. Moreover, among patients who received assisted reproductive technology, the cumulative pregnancy rate within one year after surgery in the non-chronic endometritis group (31.3 %) was significantly higher than that of the endometritis group (7.7 %) (*P* = 0.017 < 0.05), indicating that chronic endometritis may be one of the causes of infertility in patients. Improvement of the inflammation status of the endometrium, such as antibiotic treatment can improve the pregnancy rate in patients.

Chronic endometritis normally does not show symptoms. As in the 26 chronic endometritis patients diagnosed in our study, these patients do not experience abdominal or lumbosacral pain or symptoms of pelvic inflammatory disease, and their diagnosis mainly relies on the existence of plasma cells. However, because the uterine body is isolated from the immune system, the immune response of the endometrium is not strong. A low number of plasma cells in the lesion or an improperly processed specimen and interference from the granulosa cells and fibroblasts in the tissue will make it difficult to identify the plasma cells in the HE-stained sections. Even a highly experienced pathologist may miss the diagnosis. In addition, the histological changes in the endometrium at different stages can also interfere with the identification of plasma cells and the diagnosis of CE. During late menstruation or early endometrium proliferation, significant mononuclear cell infiltration, active mitosis in the stroma, stroma cell proliferation, or plasma cell-like stroma cell infiltration can occur, and significant premenstrual reactions occur during the late secretion stage and appear in the endometrial tissue. These processes can generate plasma cell-like interfering cells in the tissue that impede the diagnosis of chronic endometritis [9], thus causing high misdiagnosis and missed diagnosis rates.

Transmembrane heparin sulfate proteoglycan syndecan-1 (CD138) is the most specific indicator of plasma cells. Specific staining of CD138 with immunohistochemical methods and direct detection of the existence of plasma cells can eliminate the interference of cells with similar morphology, and these methods are expected to improve the accuracy of chronic endometritis diagnosis. In the present study, HE staining was performed on endometrial tissues of 93 patients who underwent assisted pregnancy treatment. Of these cases, no patients were diagnosed with chronic endometritis, 25 patients were diagnosed with suspected chronic endometritis, and 68 patients were diagnosed with non-chronic endometritis. Whereas results from CD138 immunohistochemical staining showed that the positive rate of CD138 immunohistochemical staining of the 25 suspected CE patients was 12 %, the CD138 positive rate of endometrial tissue of the 68 non-chronic endometritis patients was 33.8 %, and the difference was statistically significant. This result suggests that even if pathology is the gold standard for the diagnosis of chronic endometritis, due to the difficulty of identification of plasma cells, conventional pathological diagnosis has a large degree of subjectivity, resulting in cases of misdiagnosis and missed diagnosis. This is consistent with the results from Bayer-Garner et al. In chronic endometritis, the plasma cell membrane shows strong positive CD138 immunohistochemical staining, while the cytoplasm shows weak positive staining, making the cells easy to discriminate in 200x and 400x magnification fields. Thus, in suspected chronic endometritis specimens, when plasma cells cannot be identified using HE staining, CD138 immunohistochemical staining can effectively display plasma cells, making plasma cells easy to identify, thereby improving the diagnosis rate of chronic endometritis. Therefore, CD138 immunohistochemical staining can improve the diagnosis rate and diagnostic accuracy of chronic endometritis [11].

The etiology of chronic endometritis is still unclear. Previous studies have suggested that chronic endometritis may be associated with bacterial infection, intrauterine devices, intrauterine abnormalities such as endometrial polyps, endometrial ossification, assisted adjuvant radiotherapy for endometrium cancer, and endometrial lymphoid intraepithelial neoplasia-like cancer [1]. The present study suggests that a previous prolonged menstrual bleeding episode history, an abortion history or fallopian tube obstruction are associated with the pathogenesis of chronic endometritis and are independent risk factors of chronic endometritis. Therefore, for infertile patients with the above mentioned risk factors, and especially for patients who undergo assisted conception treatment, a pathological endometrial CD138 immunohistochemical detection is recommended. Thus, a clear diagnosis and timely treatment can reduce the failure rate of assisted conception and improve the reproduction prognosis of patients.

In summary, on the basis of conventional endometrial HE staining and the conventional pathological examination, the addition of CD138 immunohistochemical staining can effectively improve the morphology examination and prevent missed diagnosis and misdiagnosis of some atypical cases of CE. Patients with a previous history of prolonged menstrual bleeding episodes, a history of abortion or complications of fallopian tube obstruction may be high-risk patients for chronic endometritis. These patients should be advised to have a CD138 immunohistochemical staining examination to clarify the diagnosis and undergo timely treatment to improve their reproductive prognosis. However, multi-center, large sample-size, evidence-based medical confirmation is still lacking, and the value of CD138 immunohistochemical staining still requires further clinical studies for verification.

## Conclusions

CD138 immunohistochemistry can improve the CE diagnosis rate. A previous history of prolonged menstrual bleeding episodes, an abortion history, and a history of fallopian tube obstruction are risk factors for chronic endometritis, and a CD138 immunohistochemical examination should be advised among them.

## Abbreviations

CD138: the transmembrane heparin sulfate proteoglycan syndecan-1
CE: Chronic endometritis
HE: Hematoxylin and eosin
